# Metabolic profiling of endophytic fungi acting as antagonists of the banana pathogen *Colletotrichum musae*

**DOI:** 10.1371/journal.pone.0310442

**Published:** 2025-01-24

**Authors:** Christian Joseph R. Cumagun, Joden M. Adiova, Ruth Jakobs, Anna Rathgeb, Petr Karlovsky, Caroline Müller

**Affiliations:** 1 Molecular Phytopathology and Mycotoxin Research, University of Göttingen, Göttingen, Germany; 2 Institute of Weed Science, Entomology and Plant Pathology, University of the Philippines Los Baños, Los Baños, Laguna, Philippines; 3 Department of Chemical Ecology, Bielefeld University, Bielefeld, Germany; Wachemo University, INDIA

## Abstract

Three endophytic strains, *Phomopsis* sp., *Fusarium proliferatum*, and *Tinctoporellus epimiltinus*, isolated from various plants in the rainforest of the Philippines, were investigated regarding their ability to repress growth of the pathogenic fungus *Colletotrichum musae* on banana fruits causing anthracnose disease. An *in vitro* plate-to-plate assay and an *in vivo* sealed box assay were conducted, using commercial *versus* natural potato dextrose medium (PDA). All tested endophytes were able to significantly reduce *C*. *musae* growth compared to the control. However, the type of medium had no significant effect on lesion size of *C*. *musae* on banana. An interaction effect between fungal strain and medium could be shown. On the commercial medium, no differences between the biocontrol ability of the fungi and control treatments could be found, while there were significant differences between the fungal strains on natural medium. Lesions on banana incubated with *Phomopsis* sp. on natural medium were significantly but only slightly larger than those on banana incubated with *F*. *proliferatum*. Volatiles released by these two strains and one pathogenic strain of *F*. *graminearum* were collected using polydimethylsiloxane tubes and analyzed via gas chromatography mass spectrometry (GC-MS). Twelve volatile metabolites were detected. Benzaldehyde was the most prominent volatile emitted from the commercial and plain medium. 2-Undecanone, 2-nonanone, and phenylethyl-alcohol were detected in individual samples in both media. 1-Decanol and acoradiene were exclusive to the commercial medium, with acoradiene also being unique to *F*. *proliferatum*. Five volatileorganic compounds (VOCs)were emitted from all tested fungal species: 2-heptanone, 2-nonanone, 2-undecanone, 2-tridecanone, and phenylethyl-alcohol. Beta-acorenol was detected in *F*. *proliferatum* grown on both media. To reveal whether the medium (commercial PDA versus potato extract) affected the metabolism of the fungi, metabolic footprints were assessed *via* high performance liquid chromatography with quadrupole time of flight mass spectrometry MS (HPLC-QTOF-MS). A total of 388 metabolic signals were recorded. The intensities of 80–90% of these signals differed significantly between the two types of media. Metabolic footprints varied in response to different potato dextrose medium preparations. The two promising fungal strains may be used to reduce postharvest decay and losses in fruits.

## Introduction

Endophytic microorganisms (mostly fungi and bacteria) live inside plant tissues, realizing a unique ecological niche [1]. Endophytes are of special interest because they are a potential source of bioactive compounds for medical and commercial use [2]. It is hypothesized that metabolic interactions of the endophyte within host plant tissue modulate the production of fungal secondary (= specialized) metabolites [2]. These metabolites allow their producers to colonize plant tissue asymptomatically, compete with other microorganisms within the host [12], and protect the host from pests [3] and pathogens [4]. Therefore, screening of endophytes might be a promising approach to finding biological control agents (BCAs) and bioactive secondary metabolites that could potentially be of practical use [2] against postharvest diseases of fruits and vegetables that have attracted attention in the last two decades [5].

Bananas (genus *Musa*) are produced in more than 120 countries [6]. The main countries exporting bananas are located in Latin America (Ecuador, Costa Rica and Colombia) and Asia (especially the Philippines), thus, to reach the intended market, the bananas have to be transported over large distances [7]. Due to the long transportation, storage pathogens play an important role in reducing quality and yield of banana after harvest.

One of the key postharvest diseases of banana that occurs during storage and transport is anthracnose, caused by members of the *Colletotrichum* species complex, most importantly *Colletotrichum musae* (Berk. & M.A. Curtis) [6]. *Collelotrichum musae* infects immature bananas, while disease symptoms appear during fruit ripening [8]. The symptoms comprise sunken dark spots on the banana peel with mycelium growing inside the lesions [8]. Latent *C*. *musae* infection can additionally support the development of crown rot, which is caused by a variety of fungal species [6].

Frequent pre- and postharvest application of fungicide is necessary to prevent *C*. *musae* infection [6] but this strategy is not environmentally sustainable, and the pathogen develops resistance [6, 9]. Thus, the need for biological control alternatives is pressing [10]. The general objective of the study was to isolate and identify fungal endophytes from native and endemic plants of Mount Makiling, Los Baños, Laguna (the nearest rainforest park south of Manila) that can inhibit the growth of *C*. *musae* under in vitro and in vivo conditions. The metabolic profiles of the fungal strains in response to commercial vs natural (homemade) potato dextrose medium were also examined to gain a better overall understanding on how the medium influences the secondary metabolites, including volatile organic compounds (VOCs) and secreted metabolic fingerprints.

## Materials and methods

### Collection of plant materials

A preliminary survey of potential plant species to be sampled was done in areas surrounding a specific area called Mudspring, a boiling mud crater located on the northeast side of Mount Makiling, Los Baños, Laguna at about 270-meter altitude. A small asymptomatic tissue was collected from bark or stem of each plant using a sharp sterile blade. Collected tissue samples were then placed in a paper bag and stored in an ice-packed container while in the field. They were processed immediately in the laboratory after collection to prevent the growth of contaminants.

### Natural vs commercial potato dextrose agar (PDA)

Natural PDA was prepared by boiling 200 g of sliced, unpeeled potatoes in water for 30 min to obtain an potato infusion. The potato infusion was filtered through cheesecloth and 20 g dextrose and 20 g agar were added to the liquid to make up for 1 liter of PDA. Commercial PDA was prepared by adding 39 g of potato extract glucose agar (Roth, Karlsruhe, Germany) to 1 liter of distilled water.

### Isolation of endophytic fungi

Plant tissue samples were thoroughly sterilized following the methods described by Ezra et al. [11] to remove any surface contaminants. They were washed thoroughly in running tap water for 10 min, cut under sterile conditions into small pieces (2–3 cm) and then surface sterilized by sequential immersion in 1% sodium hypochlorite for three min, sterile water to remove traces of sodium hypochlorite for three min, and 90% ethanol (v/v) for 30 sec. Removing excess alcohol and other contaminants that possibly got away from surface sterilization was done by passing the samples briefly through the flame.

Outer surrounding tissues were removed from samples using a sharp sterile knife blade. Inner tissues were carefully excised into smaller pieces and placed on petri plates containing natural PDA supplemented with antibiotic streptomycin (3 mg/100 mL) to prevent bacterial growth. The tips of the fungal hyphae growing from tissue samples were removed and transferred to petri plates with PDA. Pure cultures were then transferred to PDA slant tubes.

### Screening of endophytes

Sampling of endophytes in the rainforest Mount Makiling, Los Baños, Laguna in the Philippines and subsequent screening against the banana pathogen *C*. *musae* was conducted. The constraint in time and resources limited the number of plants that could be collected and examined for the presence of fungal endophytes. Because of this, a strategy was used in selecting plants to be screened for the presence of promising fungal endophytes. Using a rationale for plant selection devised by Strobel and Daisy [1], a total of 42 different plant species belonging to 29 families were selected for endophyte isolation. Plants that were selected are either growing in unique environmental settings with ethnobotanical history, or are endemic. In addition, plants that have important conservation status (e.g. endangered, vulnerable, threatened) based on updated literature were considered. Part of the bark or stem of selected plants was collected, placed in a sealable plastic bag and immediately processed in the laboratory. Fungal endophytes growing in the agar-planted tissue samples were collected and then transferred to PDA plates. The top three promising fungal endophytes were selected for further tests based on their high percent inhibition values against the pathogen.

### DNA isolation and amplification

Genomic DNA extraction was performed utilizing a cetyl trimethylammonium bromide (CTAB)-based method [12]. To identify the endophytic strain A-8 and the pathogen *C*. *musae*, the ITS region was amplified with the primers ITS1 and ITS4 [13]. The endophytic strain 2.6 had been previously identified as a member of the *Phomopsis* genus by ITS region sequencing.

PCR products were purified by adding isopropanol at a final concentration of 70% (v/v), vortexing, and incubating at room temperature for 15 min. The DNA pellet was collected by centrifugation at 14,000 rpm (ca. 14,462 rcf) for 15 min, washed with 70% ethanol, dissolved in 20 μl double-distilled water and sent for sequencing to Macrogen Europe (Amsterdam, the Netherlands).

### Sequencing data evaluation

The quality of the nucleotide sequences was checked in BioEdit [14] and beginning and ending sequences with lower quality were cut off. The reverse complement sequences were generated (bioinformatics.org/sms) and then merged with the forward sequences. Conflicts were resolved by checking ABI chromatograms manually in BioEdit. The obtained sequences were blasted against the NCBI database (https://www.ncbi.nlm.nih.gov/genbank).

### Bioassays

Two different bioassays were performed to assess the antibiotic properties of VOCs produced by the fungal endophytes.

### Volatile antagonistic assay I—Plate-to-plate method

**Fig 1:**
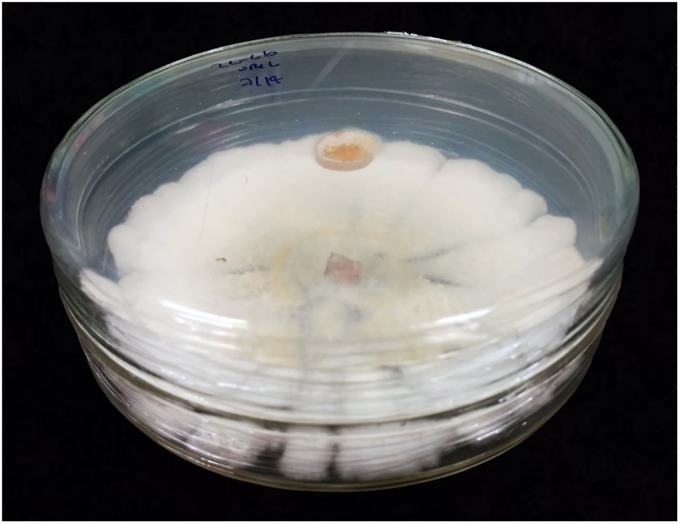
Example of the plate-to-plate set up. The plate in the back is fully covered with mycelium of an endyphytic strain 2.6 (*Phomopsis*), while on the plate in the front, the growth of the pathogen *Colletotrichum musae* is assessed. Plates are sealed together with parafilm facing each other.

A volatile antagonistic bioassay previously described by Stinson et al. [15] was performed twice. For the first trial, the endophytic strains A-8, 2.6, and 8–3 were separately grown on natural PDA petri plates until the plate was fully covered with mycelium (about 11 days at 25°C in complete darkness). A second natural PDA plate with a freshly transferred 9 mm plug of *C*. *musae* mycelium was then placed against the Petri plate containing the BCA strain. The plates were sealed together with two layers of parafilm (Fig 1). As a control, *C*. *musae* was paired against a plain natural PDA plate. Each pair was replicated five times. Growth of *C*. *musae* was assessed from day three to day seven in cm colony diameter. Data was pooled from two trials, one for the strain A-8 separately, and one for the strains 2.6 and 8–3.

For the second trial, the endophytic strains 2.6 and 8–3 as well as a *Fusarium graminearum* (Fgram) strain isolated from maize in the Philippines were separately grown on natural and commercial PDA petri plates until full coverage of the plate was reached (same conditions as above). The plant pathogen *F*. *graminearum* was used for comparison with the two endophytic strains. A second natural or commercial PDA plate with a freshly transferred 2 mm plug of *C*. *musae* mycelium was then placed against the fully covered petri plate with the respective endophytic strain or *F*. *graminearum* in the same manner as described for the first trial. Natural PDA was always placed against natural PDA, while commercial PDA was placed against commercial PDA plates. As a control, *C*. *musae* was paired against plain PDA plates. Each pair was replicated five times on natural and five times on commercial medium. Growth of *C*. *musae* was assessed from day three to day seven in cm colony diameter. Even though *C*. *musae* growth was measured up until day six, comparisons were made when the growth of the control reached full coverage of the plate. Growth inhibition was calculated using the Formula 1.

$$\left(1-\frac{lesion\;diameter\;treatment\;\left[cm\right]}{lesion\;diameter\;control\;\left[cm\right]}\right)x100$$

Formula 1. **Calculation of growth inhibition.**

Data was analyzed in R Software version 3.5.1 [16]. Comparisons were performed either with t-tests (for unequal variances, the Welch degree of freedom modification was used) or Mann-Whitney-U rank sum test. For multiple comparisons analysis of variance (ANOVA) and post hoc Tukey test were used. Normal distribution and variance homogeneity of the data were tested using the Shapiro-Wilk test and Levene test, respectively. Mixed effects analysis was performed to analyze the effects of medium and fungal strain, with subsequent likelihood ratio testing.

### Volatile antagonistic assay II—Sealed box assay

A second kind of volatile antibiotic assay was performed using organic bananas inside plastic boxes. A different set of transparent plastic boxes was used for this trial (Gerda GmbH, Schwelm, 20x20x6 cm). The plastic boxes were lined with damped paper towels. One organic unripe banana Cavendish cultivar (origin: Dominican Republic) was wiped with 70% ethanol and placed in a box, after being punctured three times, and inoculated with a *C*. *musae* spore suspension (0.5x10^6^ spores/ml) as described above. To each box two PDA plates that were fully covered with the mycelium of the respective fungal strains (10 days old) were added. The strains 2.6, 8–3 and the *F*. *graminearum* strain were used for this trial on both natural and commercial PDA. As control treatments, plain natural and commercial PDA plates were used, as well as a treatment without any added petri plates. Each combination was replicated in three boxes. Boxes were sealed, left at room temperature and inspected seven days after.

Data was analyzed in R Software version 3.5.1 [16]. Comparisons were performed either with t-tests (for unequal variances, the Welch degree of freedom modification was used) or Mann-Whitney-U rank sum test. For multiple comparison in the first trial a Kruskal-Wallis multiple comparison was used. For the second trial, a linear mixed effects analysis was performed, to analyze the effects of medium and fungal strain (R- package: *lme4* version: 1.1–19). Banana were entered as a random effect. Subsequently likelihood ratio testing was conducted of the full model with the investigated against the model without the effect. Normal distribution and variance homogeneity of the data were tested using the Shapiro-Wilk test and Levene test, respectively. Boxplots were created with BoxPlotR [17].

### Metabolomic analyses

#### Cultivation of fungi for metabolomic analyses

In order to assess the emitted VOC profiles and the secreted metabolites, the endophytic strains 8–3 and 2.6 as well as the Fgram strain were cultivated in 30 ml of natural or commercial liquid medium in a 100 ml Erlenmeyer flask. Six replicates were prepared for each strain and each medium and were incubated at 25°C in darkness for 36 hours until the mycelium was grown close to the surface of the medium. As an uninoculated control, 30 ml of natural and commercial liquid medium in a 100 ml Erlenmeyer flask were incubated in the same manner.

Inoculation was performed for the spore-producing strain 8–3 with 1 ml of a spore suspension (0.3x10^6^ spores/ml in sterile tap water). As the strains 2.6 and Fgram did not produce spores on PDA, a suspension of shredded mycelium was prepared as follows: the mycelium of one petri plate was scratched off and shredded in 20 ml sterile tap water with an ultra-turrax (IKA Werke, Staufen) for five seconds. The ultra-turrax was sterilized with 70% ethanol overnight before first time use, and for one hour in between the uses. One ml of this mycelium suspension was used to inoculate the medium flasks.

#### Preparation of polydimethylsiloxane tubes

Polydimethylsiloxane silicone tubes (PDMS, 1 mm internal diameter, 1.8 mm external diameter; Carl Roth, Karlsruhe, Germany) were prepared as described in Kallenbach et al. [18]. The PDMS tubes were cut into 5 mm long pieces and were cleaned in the following manner. First, the pieces were soaked in an acetonitrile-methanol mixture (4:1) in a glass bottle at room temperature. After 3 hours, the solvent was removed and the PDMS tubes were heated for 2 hours at 210°C and allowed to cool down to room temperature, all while being under constant nitrogen gas flow. The clean PDMS tubes were stored in glass vials until the collection of volatiles.

#### Sample preparation for analysis of volatiles

Using autoclaved stainless-steel wire (⌀ 0.2 mm, V2A 1.4301, Jena.dach.bau.technik, Navratiel, Görlitz) two PDMS tubes were inserted into each headspace above the liquid cultures and incubated for four hours to allow equilibration. Afterwards, tubes were placed in individual vials, sealed, and sent for analysis to Bielefeld University. Samples were stored at -20°C and preceding analysis, kept at room temperature for 24 hours to equilibrate.

#### TD-GC-MS analysis

The VOC samples were analyzed as described in Kallenbach et al. [18] with some modifications employing a TD-20 thermal desorption unit (Shimadzu, Kyoto, Japan) coupled to a gas chromatograph connected to a mass spectrometer (TD-GC-MS, GC 2010 Plus QP2020, Shimadzu, Kyoto, Japan). PDMS tubes were placed individually in a thermal desorption sampler tube. Desorption of compounds was performed under a helium flow of 60 ml/min for 10 min at 200°C onto a Tenax^®^ adsorbent trap at a trap temperature of -20°C. Following desorption, the trap was heated to 230°C for 10 min and the interface heat trap and line temperature were set to 230°C. A 1/10 split was used, and compounds were separated on a VF-5ms column (30 m x 0.25 mm i.d., 10 m guard column, 0.25 μm film thickness; Agilent, Santa Clara, CA, USA) with a helium flow of 1.62 ml/min. The oven temperature was set to 50°C for 5 min and subsequently ramped to 280°C at 8°C/min, where it was held for 5 min. The transfer line was held at 250°C. Mass spectra were taken in electron ionization mode at 86 eV (based on tuning result) with five scans per second in full-scan mode (30–500 *m/z*). The ion source temperature was set to 230°C and the interface temperature to 250°C. Every 20 samples an alkane standard (C8-C20, Sigma Aldrich Chemie GmbH, Steinheim, Germany) applied on a PDMS tube was measured to calculate retention indices (RI) [19]. Peaks were identified by comparing mass spectra to mass spectra of the National Institute of Standards and Technology (NIST 14) library and Flavors and Fragrances of Natural and Synthetic Compounds (FFNSC) library [20, 21]. Compounds that did not match both comparisons were named after their calculated RI. Compounds were semi-quantified based on the total ion chromatogram (TIC) of the peak. For further analysis only those compounds were considered, which were not detected in PDMS tubes (trapping medium only), had a similarity index (comparing the detected mass spectrum to the one from a library) above 70 and were detected in at least half of the samples of one group (strain x medium).

#### Secreted metabolite extraction

After retrieving the PDMS tubes, the cultures were centrifuged (12,000 rcf; 15 min) to gently separate the mycelium and the medium and therefore to prevent rupturing of the mycelium, which would lead to contamination with intracellular metabolites. The supernatant was used for the extraction of extracellular metabolites. Separation of the mycelium and the remaining medium was attempted using a Büchner funnel. Due to the preceding centrifugation step, the mycelium was firmly sedimented and could not be fully recovered. Therefore, normalization based on fungal biomass could not be performed.

Ten ml ethyl acetate with 1% (v/v) acetic acid were added to 10 ml of the supernatant and shaken for one hour at 200 rpm. The samples were then centrifuged at 4,500 rpm for 5 min and the upper phase was transferred into a new tube. One ml of the upper phase was evaporated in vacuum for about 2 hours (30°C) and the dry sample was kept at -20°C. Before analysis, the residue was redissolved in 1 ml of methanol:water (1:10) and 200 μl were transferred into HPLC vials. A quality control mixture was prepared for data normalization with an in-house Perl script by combining 50 μl aliquots of each sample. Additionally, a pool of all control samples (plain medium blanks without mycelium) was set up. The quality control was diluted 1:2, 1:4 and 1:8 with the control pool (pooled media blanks) to evaluate the normalization method.

#### HPLC-QTOF-MS analysis

The fungal extracts were analyzed via high performance liquid chromatography coupled to time-of-flight MS (HPLC-QTOF-MS) employing an Agilent 1290 Infinity II LC system coupled to an Agilent 6545 QTOF. Compounds were separated on a Varian Polaris C18 Ether column (2 x 100 mm, 3 μm particle size), which covers a wide range of polarities. A gradient of LC-MS grade methanol and distilled water (further purified by an Arium pro ultrapure water system, Sartorius, Germany) was applied. Both eluents were acidified with 0.1% formic acid. The column was eluted with a linear gradient of 95% to 0% water in methanol for 20 min, followed by pure methanol for 2 min. The flow rate was set to 0.2 ml/min and the column was heated to 35°C. After each run, the column was re-equilibrated for 7.5 min in 5% methanol. The injection volume was 10 μl. A solvent blank was measured after every fifth sample. Eluted compounds were monitored with an Agilent 6545 QTOF working with an electrospray ionization source (Jet Stream ion source, Agilent) operated in a positive ionization mode. The sheath gas temperature was set to 350°C and the flow to 11 l/min. Two TOF spectra per sec were acquired in MS1 mode from 100 to 1700 *m*/*z*. The N_2_ temperature was set to 320°C with a flow of 8 l/min and a nebulizer pressure of 35 psig. The voltages were set as follows: nozzle voltage 1,000 V, capillary voltage 3,500 V, fragmentor 175 V, and skimmer 65 V.

#### HPLC-MS/MS data analysis

Feature finding and alignment were performed employing the XCMS-based R-package MAIT 1.14.0. The dataset was visualized in Agilent Mass Profiler Professional 14.5 (MPP). Matrix features (all *m*/*z* features that were also detected in the media blanks) were removed from the data in MPP priorto normalization. The remaining features were normalized using an in-house Perl script. For further analysis only the 388 features with intensities higher than 100,000 counts in all replicates of at least one group (strain and medium) were considered. This means when comparing a specific fungal strain grown on natural medium to the same strain grown on commercial medium, the intensity had to be at least 100,000 counts in all replicates of the natural or the commercial medium. Normalized data was analyzed with an unpaired Mann-Whitney test with a p-value cut-off at 0.05 conducted for each fungal strain comparing different media. Volcano plots were generated to visually analyze the differences between treatments.

## Results

### Inhibitory activity of endophytes

A total of 80 fungal endophytes were isolated from the 42 plant species collected from Mount Makiling, and they were all antagonistic to *C*. *musae* at varying degrees of inhibition (Table 1). Of all the isolated fungal endophytes that were screened against *C*. *musae*, three strains causing the highest percent inhibition (>98%) of the colony growth of the pathogen were selected for further investigation in the present study. The strains emitted a set of VOCs that halt the growth of *C*. *musae* on banana without direct contact of fungal mycelia and they showed the highest inhibition rate on *C*. *musae* (Table 1). The rest of the strains had a wide range of pathogen growth inhibition, ranging from 5 to 93%, with a median of 45% and a mean of 42.6% (data not shown).

**Table 1:** Percent inhibition of endophytic isolates against *Colletotrichum musae*.

Isolate	Host Plant	Inhibition Rate
A-8	*Arcangelisia flava (L.) Merr.*	*9.3%*
2.6	*Tetrastigma harmandii Planch.*	*99.0%*
8.3	*Macaranga grandifolia (Blanco) Merr.*	*98.7%*
OP3	*Orania palindan (Blanco) Merr.*	*93.0%*
PI4	*Piper interruptum Opiz var. loheri (C. DC.) Quis.*	*89.0%*
BI1	*Bauhinia integrifolia Roxb.*	*85.0%*
AC	*Anamirta cocculus (L.) Wight and Arn.*	*83.0%*
PS	*Piper interruptum Opiz var. loheri (C. DC.) Quis.*	*83.0%*
PI3	*Piper interruptum Opiz var. loheri (C. DC.) Quis.*	*79.0%*
DP1	*Diplodiscus paniculatus Turcz.*	*76.0%*
ES TR6	*Tetrastigma harmandii Planch.*	*74.0%*
AF7	*Arcangelisia flava (L.) Merr.*	*71.0%*
AF6	*Arcangelisia flava (L.) Merr.*	*65.0%*
NC1	*Neotrewia cumingii (Muell.-Arg.) Pax and Hoffm*	*64.0%*
OP1	*Orania palindan (Blanco) Merr.*	*61.0%*
MB1	*Macaranga bicolor Muell.-Arg.*	*60.0%*
OP2	*Orania palindan (Blanco) Merr.*	*59.0%*
FI1	*Flagellaria indica L.*	*58.0%*
ES TR3	*Tetrastigma harmandii Planch.*	*58.0%*
AF5	*Arcangelisia flava (L.) Merr*.	*56.0%*
FP3	*Ficus aurantiacea Griff.*	*55.0%*
TS1	*Lithocarpus sulitii Soepadmo*	*55.0%*
TR4	*Tetrastigma harmandii* Planch.	*55.0%*
DL	*Diospyros blancoi A. DC*.	*54.0%*
UI1	*Ficus ulmifolia Lamk*	*52.0%*
PS2	*Myristica philippensis Lam*.	*52.0%*
Vine	*Semecarpus cuneiformis Blanco*	*52.0%*
ZI	*Zehneria japonica (Thunb.) H.Y. Liu*	*52.0%*
DM2	*Drypetes maquilingensis (Merr.) Pax and K. Hoffm.*	*51.0%*
FI2	*Flagellaria indica L.*	*51.0%*
PI2	*Saurauia latibractea Choisy*	*51.0%*
CS1	*Dillenia philippinensis Rolfe*	*50.0%*
PI1	*Piper betle L.*	*50.0%*
ES TR2	*Toono calantas Merr. and Rolfe*	*50.0%*
FP1	*Ficus aurantiacea Griff.*	*49.0%*
PB	*Piper betle L.*	*49.0%*
AF4	*Antidesma pleuricum Tul.*	*48.0%*
DP2	*Diplodiscus paniculatus Turcz.*	*48.0%*
FP2	*Ficus elastica Roxb. ex Hornem.*	*48.0%*
FS1	*Freycinetia sp.*	*48.0%*
ES TR5	*Toono calantas Merr. and Rolfe*	*48.0%*
CH2	*Citrus hystrix DC.*	*45.0%*
FS	*Ficus odorata (Blanco) Merr.*	*45.0%*
HP	*Lithocarpus sulitii Soepadmo*	*45.0%*
SC	*Semecarpus cuneiformis Blanco*	*45.0%*
II	*Ixora longistipula Merr.*	*43.0%*
AM1	*Alsomitra macrocarpa (Blume) Roem.*	*42.0%*
QS	*Saurauia latibractea Choisy*	*41.0%*
AF1	*Artocarpus ovatus Blanco*	*39.0%*
CH1	*Citrus hystrix DC.*	*38.0%*
An	*Antidesma pleuricum Tul.*	*37.0%*
SP	*Syzygium calubcob (C.B. Rob.) Merr.*	*37.0%*
DM1	*Drypetes maquilingensis (Merr.) Pax and K. Hoffm*.	*36.0%*
MO	*Myristica philippensis Lam.*	*36.0%*
AB	*Agelaea borneensis (Hook.f.) Merr.*	*31.0%*
CS2	*Ficus septica Burm.f.*	*31.0%*
BI2	*Bauhinia integrifolia Roxb.*	*26.0%*
CH4	*Citrus hystrix DC.*	*26.0%*
DZ	*Dimorphocalyx luzoniensis Merr.*	*26.0%*
FS2	*Freycinetia sp.*	*26.0%*
MS1	*Malaisia scandens (Lour.) Planch*	*26.0%*
SE	*Schefflera elliptica (Blume) Harms*	*25.0%*
ML	*Miliusa vidalii J. Sinclair*	*23.0%*
MS2	*Malaisia scandens (Lour.) Planch*	*22.0%*
MB2	*Macaranga bicolor Muell.-Arg*.	*20.0%*
CH3	*Elaeocarpus monocera Cav.*	*18.0%*
MC3	*Momordica cochinchinensis (Lour.) Spreng*.	*18.0%*
EN BI3	*Bauhinia integrifolia Roxb.*	*16.0%*
EN AZ	*Alocasia zebrina C. Koch. and Veitch*.	*15.0%*
EN PS3	*Myristica philippensis Lam*.	*15.0%*
EN NC2	*Neotrewia cumingii (Muell.-Arg.) Pax and Hoffm*	*15.0%*
EN DP3	*Elaeocarpus monocera Cav*.	*14.0%*
EN DD	*Diospyros blancoi A. DC.*	*13.0%*
EN AF3	*Artocarpus ovatus Blanco*	*11.0%*
EN CM	*Cinnamomum mercadoi Vidal*	*11.0%*
EN PS1	*Miliusa vidalii J. Sinclair*	*10.0%*
EN FP	*Ficus elastica Roxb. ex Hornem.*	*9.0%*
EN MC2	*Momordica cochinchinensis (Lour.) Spreng*.	*9.0%*
EN DPh	*Dillenia philippinensis Rolfe*	*7.0%*
EN AM2	*Alsomitra macrocarpa (Blume) Roem*.	*5.0%*

The endophytic strain A-8 was identified as *Tinctoporellus epimiltinus* species (accession noPP825350). The strain 2.6 was identified as a *Phomopsis* species (accession no OR999064) and sequencing of the histone H3 gene was performed to obtain identification on species level to *Diaporthe arecae*. The strain 8.3 was identified as *Fusarium proliferatum* (accession no PP034522) by sequencing of the TEF gene.

### Volatile activities in plate-to-plate method (bioassay I)

**Fig 2:**
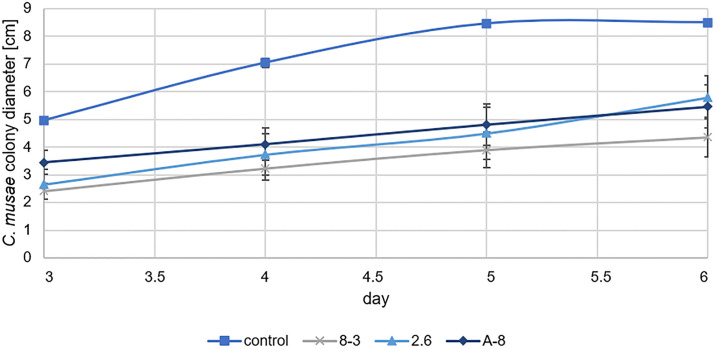
*Colletotrichum musae* growth on natural PDA against three endophytic strains in a plate-to-plate set up. A freshly inoculated PDA plate with *C*. *musae* was placed against a plate fully covered with the respective endophytic strain (means of n = 5). Control growth was measured against plain PDA. Error bars indicate the standard error.

On day five, the control growth of *C*. *musae* reached the borders of the petri dish (8.5 cm ± 0, mean ± SE, Fig 2), and this day was used to draw a conclusion about the growth-reducing activities of the produced VOCs by the endophytic strains. All tested endophytes were able to significantly reduce *C*. *musae* growth compared to the control (one-way ANOVA with Tukey HSD test with p ≤ 0.05).

The strain 8–3 was the most potent strain in reducing *C*. *musae* growth, where one strain differed strongly from all the others. This amounts to a growth reduction of about 4.6 cm (one-way ANOVA, Tukey test p = 0.001). The strain 2.6 produced VOCs with a growth reduction of 4.0 cm (one-way ANOVA, Tukey test p = 0.004). VOCs produced by the strain A-8 were able to reduce *C*. *musae* colony diameter by 3.6 cm (one-way ANOVA, Tukey test p = 0.008) to 4.8 cm ± 0.7. No significant differences between the performances of the potential BCAs could be established. Growth inhibition was calculated and ranged between 54% (8–3) and 43% (A-8), with 2.6 at 47%.

**Fig 3:**
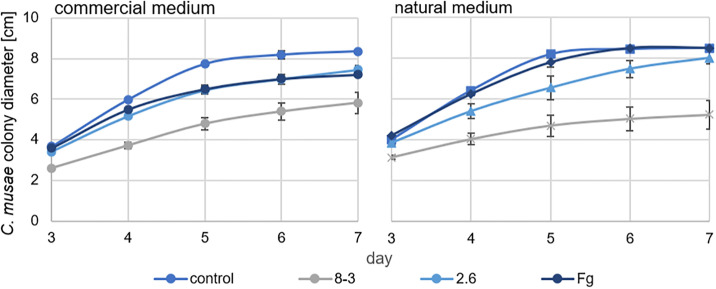
*Colletotrichum musae* growth against different endophytic strains in a plate-to-plate set up on commercial and natural medium. A freshly inoculated PDA plate with *C*. *musae* was placed against a plate fully covered with the respective endophytic strain (n = 5), tested on both commercial and natural PDA. Control growth was measured against plain PDA. Error bars indicate the standard error.

During the progression of the practical work, the stock culture of the endophytic strain A-8 lost its viability. The second trial was therefore performed without this strain, but a second *Fusarium* species was included as a negative control. *C*. *musae* control growth was slower during this trial and did not reach full coverage of the plates on commercial medium during the trial (8.4 cm ± 0.1). On natural medium, the *C*. *musae* control growth reached full coverage of the plates on day six (8.5 cm ± 0.1). Therefore, for comparison of volatile antibiotic activity, the measurements from day six were used. A one-way ANOVA with a post-hoc Tukey HSD test revealed that only the strain 8–3 could statistically significantly reduce growth of *C*. *musae* on this plate-to-plate assay (Fig 3). A difference between the two media could not be safely established (p = 0.059).

VOCs of 8–3 amounted to a growth reduction of 2.8 cm on the commercial medium (growth inhibition of 34%) and 3.4 cm on natural medium (growth inhibition of 40%). There was no significant difference between the performance of 8–3 on natural versus commercial medium. Comparisons of each fungal strain on both media did not show statistically significant differences in growth reduction on day six. Additionally, no significant differences could be shown between the plain medium and the medium containing Fgram. Statistically significant differences between the plain medium and the strain 2.6 could only be shown when medium was not taken into account (p = 0.019).

Control growth of *C*. *musae* was significantly different on days four and five of the measurement (Table 2). For the two endophytic strains 8–3 and 2.6 there were no significant differences except on the first day of measurement, day three. Only for Fgram consistent significant differences between the growth on the commercial and the natural medium could be shown. In the case of significant differences, the colony diameter of *C*. *musae* was always lower on the commercial medium than on the natural one.

**Table 2:** Mean values of *Colletotrichum musae* colony diameter against different fungal strains in a plate-to-plate set up.

Fungal strain	Day	Mean colony diameter (± SE) [cm]		Significant difference
		Commercial medium	Natural medium	
Control	3	3.7 (± 0.1)	4.0 (± 0.2)	p = 0.242 n.s.
	4	6.0 (± 0.1)	6.5 (± 0.2)	p = 0.0343 *
	5	7.7 (± 0.1)	8.2 (± 0.2)	p = 0.028 *
	6	8.2 (± 0.2)	8.5 (± 0.1)	p = 0.239 n.s.
	7	8.4 (± 0.1)	8.5 (± 0)	p = 0.180 n.s.
2.6	3	3.4 (± 0.1)	3.9 (± 0.2)	p = 0.025 *
	4	5.2 (± 0.1)	5.4 (± 0.4)	p = 0.548 n.s.
	5	6.5 (± 0.2)	6.6 (± 0.6)	p = 0.850 n.s.
	6	7.0 (± 0.2)	7.5 (± 0.4)	p = 0.345 n.s.
	7	7.5 (± 0.2)	8.0 (± 0.3)	p = 0.175 n.s.
8–3	3	2.6 (± 0.1)	3.2 (± 0.1)	p = 0.001 **
	4	3.7 (± 0.2)	4.1 (± 0.3)	p = 0.353 n.s
	5	4.8 (± 0.3)	4.7 (± 0.5)	p = 0.897 n.s
	6	5.4 (± 0.4)	5.1 (± 0.6)	p = 0.630 n.s
	7	5.9 (± 0.5)	5.2 (± 0.7)	p = 0.497 n.s
Fgram	3	3.6 (± 0.1)	4.2 (± 0.1)	p = 0.001 ***
	4	5.5 (± 0.1)	6.3 (± 0.1)	p = 0.001 **
	5	6.5 (± 0.4)	7.8 (± 0.2)	p = 0.024 *
	6	7 (± 0.4)	8.5 (± 0)	Not available
	7	7.2 (± 0.4)	8.5 (± 0)	Not available

A freshly inoculated PDA plate with C. musae was placed against a plate fully covered with the respective endophytic strain (n = 5), tested on both commercial and natural PDA. Control growth was measured against plain PDA. Colony diameter of different strains at a certain day was compared using a student’s t-test or a Mann-Whitney-U rank test. Asterisks indicate p-values < 0.001 ***, < 0.01 **, < 0.05 *,; n.s. = not significant, not available = no statistical test conducted, since variances were not equal and data was not normally distributed, SE = standard error.

### Volatile activities in sealed box assay (bioassay II)

**Fig 4:**
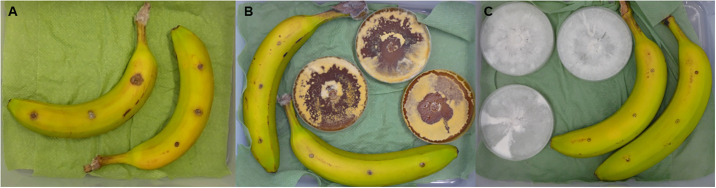
Pictures of sealed box pre-trial. Bananas were punctured with a disposable needle (Erosa, 0.07x32 mm) and artificially inoculated with 10 μl of *Colletotrichum musae* spore suspension (0.5x10^6^ spores/ml). Bananas were incubated inside a closed plastic box, with or without open petri plates of endophytic fungal strains. A: without any petri plates, B: with 2.6 (17-day old culture), C: with A-8 (10-day old culture).

In a test run of the trial using the sealed box assay, only two endophytic strains were tested (Fig 4), A-8 and 2.6. All plates were fully covered with mycelium, but plates 2.6 were 17 days old at the start of the trial, while A-8 plates were 10 days old. After seven days, no sign of infection was visible on bananas that were incubated with the A-8 strain, only the puncture wounds were visible (mean: 0.07 cm ± 0) (Fig 4C). On bananas incubated with 2.6 small lesions were visible (0.3 cm ± 0.1), while on control bananas incubated without fungi present, the biggest lesions were observed (1.0 cm ± 0.2). Differences were statistically analyzed with Kruskal-Wallis multiple comparison [22], which could establish a significant difference between the control treatment and A-8.

**Fig 5:**
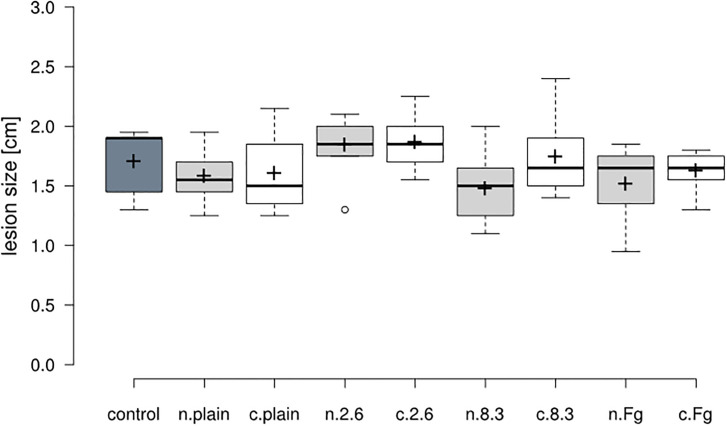
*Colletotrichum musae* lesion size on banana after incubation with different fungal strains in closed plastic boxes. Measurement 7 days after inoculation with a 0.5x10^6^ spores/ml suspension of *C*. *musae*. Control: no PDA plates inside the box, letters in front of the fungal strains indicate growth on commercial (c.) or natural (n.) medium dark grey: control (no plates), light grey (natural), white (commercial). Solid lines indicate medians, plus signs means, open circles outliers, boxes the interquartile ranges and whiskers extend up to 1.5 times the interquartile range.

**Fig 6:**
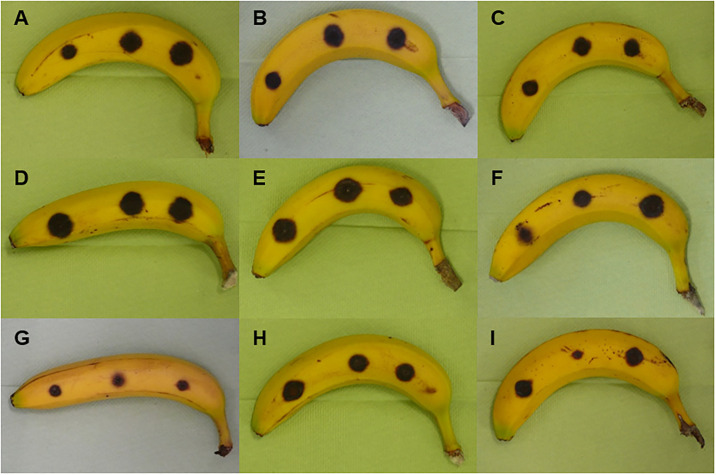
Pictures of bananas with lesions after each treatment, tested in the volatile box assay 2. Measurement 7 days after inoculation with a 0.5x10^6^ spores/ml spore suspension of *Colletotrichum musae*. A: no medium, B: plain commercial, C: plain natural, D: 2.6 (*Phomopsis*) commercial, E: 2.6 natural, F: 8–3 (*F*. *proliferatum*) commercial, G: 8–3 natural, H: Fg (*F*. *graminearum*) commercial, I: Fg natural.

The type of medium had no statistically significant effect on lesion size of *C*. *musae* on banana (χ^2^ (1) = 2.89, p = 0.089). No interaction between fungal strain and medium was found (χ^2^ (3) = 2.74, p = 0.433) (Fig 5). On the commercial medium, no differences between the performance of the fungi and control treatments were found (χ^2^ (3) = 5.469, p = 0.141), while there were statistically significant differences between the growth of the fungal strains on natural medium (χ^2^ (3) = 8.297, p = 0.0403). Lesions on banana that were incubated with 2.6 on natural medium were statistically significant but only slightly bigger than those incubated with 8–3 (p = 0.014) and Fgram (p = 0.032). No significant differences between the ability of fungi to produce VOCs that could inhibit *C*. *musae* growth could be observed when assessing the difference between the two media. Mean lesion size did not significantly differ between the different media for each fungal strain (Fig 6).

### Detected VOCs

Twelve VOCs could be detected in the test samples. Three VOCs were unique to the plain medium, both in the VOC profiles of the commercial and in the natural medium (Table 3). These three VOCs were putatively identified as benzaldehyde and its derivates benzeneacetaldehyde and 2,4-dimethylbenzaldehyde. Benzeneacetaldehyde could also be found in the headspace of *F*. *proliferatum* in one sample. The most prominent VOC in the plain commercial medium was benzaldehyde, while the highest signal in the plain medium came from benzeneacetaldehyde. Other VOCs found in the plain medium were 2-undecanone detected in one sample in the natural medium and 2-nonanone as well as phenylethyl-alcohol detected each only once in the commercial medium. The fungus *F*. *proliferatum* is capable of producing bioactive chemicals, which are crucial for promoting plant growth [23]. Two VOCs could only be detected in the commercial medium. 1-Decanol was only found in the headspace of *Phomopsis* growing on the commercial medium. Similarly, acoradiene could only be found in the VOC profile of *F*. *proliferatum* growing on the commercial medium. None of the detected VOCs were unique to the natural medium.

**Table 3:** Presence and comparison of peak area of volatiles detected in the headspaces of the endophytic strains (2.6 –*Phomopsis*, 8–3 –*F*. *proliferatum*) and *F*. *graminearum*, as well as the plain media.

Volatile compound	Media type	2.6 (*Phomopsis* sp.)	8–3 (*F*. *proliferatum*)	*Fgram* (*F*. *graminearum*)
Benzaldehyde	**Commercial ↑** ***	n.d.	n.d.	n.d.
Benzeneacetaldehyde	Natural ↑ **	n.d.	n.d.^1c^	n.d.
2,4-dimethyl-benzaldehyde	Natural ↑ *	n.d.	n.d.	n.d.
2-heptanone	n.d.	Commercial ↑ ***	C ↔ N	**Natural** ↑ **
2-nonanone	n.d.^1a^	**Commercial** ↑ ***	C ↔ N	**Natural** ↑ ***
2-undecanone	n.d.^1b^	Commercial ↑ ***	C ↔ N	**Natural** ↑ ***
2-tridecanone	n.d.	Detected only in commercial	C ↔ N	C ↔ N
Phenylethyl-alcohol	n.d.^1a^	Commercial ↑ *	**Commercial** ↑ **	**Commercial** ↑ **
1-octanol	n.d.	**Commercial** ↑ **	n.d.^1b^	n.d.^2c^
1-decanol	n.d.	Detected only in commercial	n.d.	n.d.
Acoradiene	n.d.	n.d.	Detected only in commercial	n.d.
Beta-acorenol	n.d.	n.d.	**Commercial** ↑ *	n.d.

Volatiles were trapped for 4 hours with PDMS tubes (n = 6 per medium) and analyzed via TD-GC-MS. Compounds that were detected in all fungal strains are underlined. Natural/ commercial**↑** indicates statistically significant higher peak that are in the respective medium (fold changes higher than 2 are highlighted in bold), “C ↔ N” represents no significant differences. Compounds that could not be detected in the respective strain are designated with n.d. (not detected), number indices present number of samples the compound was found in, when it was not enough to complete statistical analyses, letter indices show whether it was only detected in a = commercial medium, b = natural medium c = in both. Peak area was compared using a student’s t-test, with the Welch degree of freedom modification if necessary. Asterisks indicate p-values < 0.001 ***, < 0.01 **,< 0.05 *.

Five of the detected VOCs were present in the headspace of all of the tested fungal species (underlined compounds). These VOCs were putatively identified as 2-heptanone, 2-nonanone, 2-undecanone, 2-tridecanone and phenylethyl-alcohol. Acoradiene and beta-acorenol could only be detected in the VOC profiles of *F*. *proliferatum*. While acoradiene was only found when *F*. *proliferatum* grew on commercial medium, beta-acorenol was found under both conditions. More VOCs could be detected when the endophytic strain was grown on the commercial medium. 2-Tridecanone and 1-decanol were only found when the strain was grown on commercial medium. The other VOCs were all found in higher abundance emitted from the commercial medium (Table 3). 2-Nonanone and 1-octanol both had fold changes higher than 2 when comparing the growth on commercial with growth on the natural medium for *Phomopsis*. The peak area of 2-nonanone in the commercial medium was significantly higher than the peak area in the headspace of the natural medium (t-test, p < 0.001). The detected peak area of 1-octanol was four-times higher on the commercial medium compared to the natural medium (t-test, p = 0.001).

For four of the VOCs (2-heptanone, 2-nonanone, 2-undecanone and 2-tridecanone) no significant differences in peak area could be found when comparing the headspace of *F*. *proliferatum* grown on commercial and on natural medium. Acordiene could only be detected when grown on commercial medium, as mentioned above. Additionally, phenylethyl-alcohol and beta-acorenol had higher average peak areas when *F*. *proliferatum* was grown on commercial medium, both with fold changes higher than 2 (2.1 and 3.7, respectively). The pathogenic *F*. *graminearum* strain emitted more volatiles when growing on natural medium, in contrast to the two endophytic strains (Table 3). 2-heptanone, 2-nonanone and 2-undecanone showed approximately 2-fold changes in peak area comparing growth on natural to commercial medium. 2-nonanone was the most prominent volatile compound in terms of peak size, which was consistent with the other fungal strains.

### Metabolic fingerprints

After excluding all signals from the media, a total of 388 fungi-associated *m*/*z* features with chromatographic peak areas higher than 100,000 in the raw data in all replicates of at least one group were detected. In the following, the term group denotes one fungal strain on one medium (strain x medium).

**Fig 7:**
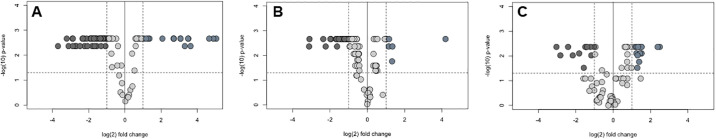
Volcano plot of metabolic footprints of (A) endophytic strain 2.6 (*Phomopsis*), (B) 8.3 (*Fusarium proliferatum*) and (C) Fgram (*Fusarium graminearum*) grown in commercial and natural medium. Those *m*/*z* features were plotted, which were in present in all replicates (N = 3) of one medium with intensities higher than 100,000 (in raw data) and were missing at most in one replicate of the other medium. For each feature, the negative log10 of the p-value (Mann-Whitney test) is plotted against the log2 of the fold change between the commercial and natural medium. Cut-off lines represent fold changes of 2 compared to the other medium and statistical significance (p < 0.05). Features with statistical significance and with log2 fold changes higher than 1 or lower than -1 are highlighted in medium grey (fold changes > 1, in higher amounts in commercial medium) or in dark grey (fold changes < -1, in higher amounts in natural medium).

In the metabolite extract of the medium of 2.6 (*Phomopsis*), 175 features with intensities higher than 100,000 in the raw data in all replicates of either commercial and/or natural medium were found. The number of features, which were present in both groups in all replicates of one group and in at least one replicate of the second group was 132. Of those 43 features, which were only present in one group, 18 features were found only in the commercial medium and 25 features in the natural medium. For comparison between the two media (Mann-Whitney-Test), those features were selected, which were present in all replicates of one group and missing at most in one replicate of the other group. The 102 features that met these criteria were used to create a volcano plot (Fig 7A). Sixty-six features (64.7%) were detected in higher amounts in the natural medium, compared to 26 features (25.5%) detected in higher amounts in the commercial medium. Only 10 of these features (9.8%) did not differ significantly in abundance between the media. The fold changes of the significantly differing features were also investigated. Fold changes below 2 were considered marginal. Therefore, log2 values of the fold changes had to be higher than 1 (higher contents in commercial) or lower than -1 (higher contents in natural). Out of the 102 features selected for analysis, 71 had a log2 fold change higher than 1 or lower than -1. Fifty-four features had a log2 fold change of lower than -1 and were found in at least twice the amounts in the natural medium. However, 17 features showed log2 fold changes higher than 1 and were detected in at least twice the amounts on the commercial medium.

In the metabolite extracts of the media of 8–3, 136 features with intensities higher than 100,000 in the raw data in all replicates of one group were detected. Of these, 115 features were present in at least one replicate in the other group as well. Twenty-one features only appeared in one medium and were absent in the other: 11 were found only in the commercial and 10 only in the natural medium. For the creation of the volcano plot, 103 features were selected, which were present in all replicates of one medium, only missing at most one replicate in the other one (Fig 7A). Ninety-one of those 103 features differed significantly from each other (88.4%). Seventy features (68.0%) were detected in higher amounts in the natural medium, while 20 features (19.4%) were found in higher amounts in the commercial medium. Thirteen features were not significantly different (12.6%). One third of the 103 features selected for analysis showed a log2 fold change higher than 1 or lower than -1. Of the 34 features, 28 displayed fold changes of 2 or more in the natural medium in contrast to 6 features with fold changes of 2 or more in the commercial medium.

In Fgram medium metabolite extracts, 134 total features with intensities higher than 100,000 in the raw data in all replicates of one group were found. Out of these, 103 features were detected in at least one replicate in the other medium, too. 31 features were only found in one of the media, 26 were found only in commercial medium, and 5 only in the natural medium. 82 features were detected in both groups, in all replicates in one group and missing at most one replicate in the other one. A volcano plot was generated with these 82 features (Fig 7C). About half of these features did not differ significantly for the two media (42 features, 51.2%). Of the other half, 24 features (29.3%) were found in higher amounts in the commercial medium and 16 (19.5%) in the natural medium. Of the 40 features that did significantly differ, 27 displayed a log2 fold change higher than 1 or lower than -1. In this case, they were more even; 14 features were found at least twice as much in the commercial medium and 13 in the natural medium.

## Discussion

In this project, several endophytic strains isolated from various plants in the rainforest of the Philippines (Mount Makiling, Los Baños, Laguna) were investigated regarding their ability to repress growth of the pathogenic fungus *C*. *musae* on banana. For this purpose, two different volatile antibiotic bioassays were conducted, an *in vitro* plate-to-plate assay as well as an in vivo sealed box assay. Additionally, the metabolic profiles of the investigated strains in response to commercial (Carl Roth GmbH) versus natural (home-made) potato dextrose medium were examined to gain a better overall understanding of how the medium influences secondary metabolism. VOC profiles as well as the composition of the secreted metabolic fingerprints were studied to determine if these are possible mechanisms of biocontrol activity of the endophytic fungi against *C*. *musae*.

In the plate to plate method, there was no direct physical contact between the fungal endophyte and the target pathogen. Any inhibition of mycelial growth of the target pathogen could thus be solely attributed to the VOCs produced by the fungal endophyte. Both *Phomopsis* and *F*. *proliferatum* significantly reduced the growth of *C*. *musae* in vitro and anthracnose infection on bananas. The medium had no significant effect on the lesion size but it did affect secondary metabolites produced by the fungi. The VOCs produced by the fungal endophyte likely have antifungal properties that disrupt the growth or metabolism of the pathogen. This could involve altering the pathogen’s cellular processes or damaging its cell structure. Using VOCs from fungal endophytes could be a sustainable and environmentally friendly way to manage plant pathogens, reducing the need for chemical fungicides.

For metabolomic analyses, the emitted volatile profiles and the secreted metabolites were assessed in liquid medium. To ensure uniform growth of the fungi inside the liquid medium, inoculation of the medium with a spore suspension turned out to be ideal. Since neither the *Phomopsis* strain nor the *F*. *graminearum* showed sufficient spore production, mycelium scraped off a plate and shredded with an ultra-turrax was used as an inoculum. This proved to work very well, as the cultures showed satisfactory growth, comparable to the growth of cultures inoculated with a spore suspension. However, a direct comparison of the amount of inoculum of spore suspensions and shredded mycelium is not possible. Trapping of VOCs on PDMS tubes as described in Kallenbach et al. [18] worked effectively. PDMS tubes provide an inexpensive and robust alternative to solid-phase microextraction (SPME), which use essentially the same material but are more costly [18, 24]. For testing which VOC would be in the end responsible for the antibiotic effects, individual commercially available VOCs would need to be tested.

While the pathogenic *Colletotrichum* strain could safely be identified as *C*. *musae* with the help of the sequencing data, this was not the case for the other two strains 2.6 (*Phomopsis*) and A-8. Species identification of 2.6 by the partial histone H3 gene sequencing failed, as no clear match was found. The sequenced ITS region of A-8 showed the best match for a strain, which was identified as *Tinctoporellus epimiltinus*. While a confident identification could not be made with these results, it seems that the strain belongs to the Basidiomycetes of the class Agaricomycetes, as all matches belong to this class. Agaricomycetes are common wood-decayers [25], and it is quite likely that this endophyte was isolated from its host as a latent saprophyte. Additionally, this indicates that the previous identification as a *Trichoderma* strain had to be the result of contamination. As this A-8 strain was identified as a Basidiomycete, it would have been especially interesting to compare it to the other two endophytic strains 2.6 and 8–3, since they are both Ascomycetes. Crous and Groenewald [26] have hypothesized that fungal plant pathogens, which are host-specific, are capable of colonizing non-host tissue without causing disease. By doing so, the pathogens are able to disperse and increase the probability to find their true host. This so-called “pogostick hypothesis” provides a possible explanation as to why the fungal species investigated in this project have all been related to plant disease or wood decay [25, 27, 28].

All strains were shown to produce VOCs that could reduce *C*. *musae* growth at least one point during the course of this project. Strobel et al. [29] reported lethality of *C*. *musae* in response to the VOCs of *M*. *albus*. Nevertheless, lethality could not be demonstrated in response to the VOCs of any of the investigated fungal strains in these bioassays, as *C*. *musae* growth was not entirely stopped. However, its growth was slowed down. This has implications for possible further use, since the lethality of VOCs would be preferred over growth-reducing characteristics as in the case of *M*. *albus* [29]. The first plate-to-plate assay on natural medium revealed growth reduction of *C*. *musae* against all three endophytic strains 8–3, 2.6 and A-8 compared to a control against plain PDA. When the trial was repeated on both media, only 8–3 was shown to significantly reduce growth of *C*. *musae* on the day the control reached full growth (day six). No differences in performance could be shown in the different media on this day. Nonetheless, it seemed like *C*. *musae* grew slower on the commercial medium and *F*. *graminearum* faster on the commercial medium. This indicates that the media affect the growth rate of the fungal strains differently. The loss of efficacy can indicate that the strains are not stable over periods of sub-culturing. Prášil and Šašek [30] showed that various Pyrenomycetes lost their antibiotic activity when experiments were repeated over time, especially when the cultures were repeatedly re-inoculated.

The first sealed box assay looked very promising, as the bananas incubated with A-8 did not show any lesions at all, and those bananas incubated with 2.6 had only small lesions. However, these results must be interpreted with caution. The VOCs of 2.6 and A-8 were each only tested in one box, therefore lesions (data points) are not independent of each other, and statistical analysis is problematic. This initial trial was performed to mainly validate the set up and subsequently, the trial was repeated with a higher number of replicates (three boxes per treatment) and considering both media. As a higher amount of boxes was necessary, different boxes were used, which were available in the required quantity. These proved to be not airtight when closed, as the distinct smell of banana could be observed when standing in front of them after the incubation period. The effect of the first trial could not be reproduced. All inoculated bananas developed lesions, and these showed approximately the same size. Statistically significant differences could only be shown for bananas incubated with 2.6 on natural medium, these bananas had slightly bigger lesions than all other treatments on natural medium. There are several explanations for this result. First of all, as with the plate-to-plate assay, the stability of antibiotic activity cannot be guaranteed over several sub-culturing cycles and long storage periods [30]. Secondly, it can be assumed that the volatiles responsible for the growth-reducing effect need to accumulate to a certain concentration in the atmosphere to cause the effect. Additionally, the first trial was performed during summer (August 2018) and the second trial during fall (October 2018), and as there is no air conditioning system inside the laboratory, room temperature likely differed substantially. Therefore, infection conditions were not the same, which could impact the results, as temperature was not accounted for in the set up. Moreover, the boxes of the second trial were contaminated with mites, which could also have affected the outcome. Additional investigations with truly air-tight containers are necessary to draw any more conclusions about the biological potential of the volatiles.

Generally, comparisons between the VOCs of the different fungal strains should be made with caution, as no normalization was performed. Twelve volatiles were found over all profiles. Three of these volatiles were only found in the plain medium and not in any of the samples where fungi were grown. These volatile compounds were putatively identified as benzaldehyde, benzeneacealdehyde and 2,4-dimethylbenzaldehyde. Although there have been reports, where fungi produce benzaldehyde [31], these are also common VOCs of raw and processed potato [32, 33]. The five volatiles, which were found in the headspace of all tested fungal species, were putatively identified as 2-heptanone, 2-nonanone, 2-undecanone, 2-tridecanone and phenylethyl-alcohol. Overall fungal strains and media, 2-nonanone was always the volatile with the highest concentration detected, followed by 2-undecanone. All of them have been connected to antifungal properties [34–39]. Specifically, the 2-nonanone vapor was used to control of decay in modified-atmosphere packages of sliced apples [40]. This is especially interesting, as they were also found in the *F*. *graminearum* strain, which was not shown to produce VOCs with growth-reducing effects toward *C*. *musae* during this project. Possibly, the VOCs were produced in lower quantity, so exposure did not lead to a growth-reducing effect. Additionally, they might need to be released in certain ratios to each other, as usually, a mixture of volatiles is responsible for the full antibiotic activity of the fungi, not just a single one [29].

None of the volatile compounds were unique for the two endophytic strains 2.6 (*Phomopsis*) and 8–3 (*F*. *proliferatum*). Thus, although there were volatiles specific to a certain fungal strain or medium, none of them could be related to the antibiotic activity of the strains. This fact also points towards the hypothesis that the VOCs need to occur in defined ratios to each other to develop their antibiotic potential. In addition, it has to be noted that the antibiotic volatile bioassays were performed with the fungal strains grown on solid medium, while VOC analysis was performed on the fungal strains grown in liquid medium, while still being submerged. This was done to get the best reproducible results from within groups (strain x medium) and to be able to easily extract secreted metabolites from the medium. However, this procedure has several consequences. First of all, it is not known if the strains retain their antibiotic activity while being submerged. As Prášil and Šašek [30] have shown, antibiotic activity of fungi can decrease when transferred from static to submerged cultures. Secondly, fungal culture age can play an important role in the production of VOCs [41]. Trapping of VOCs was performed 1.5 days after inoculation of liquid medium, while biological activity was assessed around 10 days after inoculation of the solid petri plates, when they were fully covered. This leads to the assumption that there might be no connection between the VOCs identified in this study and the biological activity. It might be better to trap VOCs of fungal cultures grown on solid medium at later stages.

There were noticeable differences between the media with regard to the metabolic data. In both endophytic strains 2.6 and 8–3, the growth on natural medium yielded in more features being upregulated. In contrast to that, in the pathogenic *F*. *graminearum* strain, of those features that were significantly different, there were more upregulated in the commercial medium. About half of the features did not show a significant difference between the media in *F*. *graminearum*, while these features only made up around 10% in the two endophytic strains. Possibly, the endophytes are more affected by the differences in the media, but this could also be a random observation, since only three strains were tested. This observation may need to be tested with more species. It is obvious that there are differences between the metabolic profiles, when fungi are grown on the two media, however, the nature of these differences remains to be investigated in further detail.

It is well described in the literature, that VOC production can be affected by medium [42] i.e. by available nutrients. Also, the production of secondary metabolites and pigmentation in general can be affected by the concentration of trace elements in the medium [43, 44]. Contents of trace elements such as copper have been shown to vary greatly between batches, in both commercial and natural PDA, and to affect morphology and pigmentation of fungi [44]. Although the authors showed that copper contents can also differ largely in PDA batches that were prepared in the laboratory (natural PDA), potatoes cultivated organically had higher copper contents than those cultivated conventionally. Griffith et al. [44] hypothesize, that contents of copper might be higher in organically produced potato, because they are more likely to have been treated with Bordeaux mixture (a mixture of CuSO_4_ and Ca(OH)_2_) as an alternative to synthetic fungicides, which ensures a high copper supply in the soil. Organically produced potatoes are more often used to prepare media to avoid fungicide residues. In the case of patulin production being suppressed on commercial PDA, it has been shown that manganese is a vital prerequisite for its synthesis and that supplementation of PDB with manganese has a great impact on its production [43, 45]. Additionally, anecdotal evidence has been presented by O’Brien et al. [46] that commercial PDA from certain companies is better suited for the production of patulin than PDA preparations from other companies.

## Conclusion

This study highlights the potential use of endophytic fungal strains collected from the rainforest in the Philippines in agricultural practices to combat a fungal disease in bananas. The three endophytic strains (*Phomopsis sp*., *Fusarium proliferatum*, and *Tinctoporellus epimiltinus*) were effective in repressing the growth of the pathogenic fungus *Colletotrichum musae*, which causes anthracnose disease in bananas. The medium type did not affect the size of lesions caused by *C*. *musae* on bananas. Significant differences in biocontrol efficacy were observed on natural medium, but not on commercial medium. Lesions were slightly larger with *Phomopsis* sp. compared to *F*. *proliferatum* on natural medium. VOC profiles differed partly between the strains. HPLC-QTOF analysis revealed that 80–90% of metabolic signals varied significantly between the fungi growing in the two media, indicating a change in fungal metabolism. The study suggests that *Phomopsis* sp. and *F*.*proliferatum* could serve as biological control agents to reduce fruit postharvest losses, with further research needed to understand the metabolic changes observed.

## Supporting information

S1 Table Chemicals, reagents, primers and equipment. (DOCX)

S1 File Colony diameter plate experiment. (XLSX)

S2 File Lesion size box experiment. (XLSX)

S3 File Volatile profile. (DOCX)
