# Supplementary material for: Metabolic profiling of endophytic fungi acting as antagonists of the banana pathogen Colletotrichum musae
Source: PLoS One. 2025 Jan 24;20(1):e0310442. doi: 10.1371/journal.pone.0310442 (PMC11760007; doi:10.1371/journal.pone.0310442)
Supplement: S1 Table — (DOCX) [file pone.0310442.s001.docx]

*Table 1.* **MAIT parameters for feature finding***.*

| **Parameter** | **Value** | **Parameter** | **Value** | **Parameter** | **Value** |
| --- | --- | --- | --- | --- | --- |
| snThres | 10 | mzWidGroup | 0.25 | fwhm | 30 |
| Sigma | 2.123322575 | filterMethod | centWave | family1 | gaussian |
| mzSlices | 0.3 | rtStep | 0.03 | family2 | symmetric |
| retcorrMethod | loess | nSlaves | 0 | span | 0.2 |
| groupMethod | density | ppm | 10 | centWave peakwidth1 | 10 |
| bwGroup | 3 | minfrac | 1 | centWave peakwidth2 | 25 |
