# Supplementary material for: Metabolic profiling of endophytic fungi acting as antagonists of the banana pathogen Colletotrichum musae
Source: PLoS One. 2025 Jan 24;20(1):e0310442. doi: 10.1371/journal.pone.0310442 (PMC11760007; doi:10.1371/journal.pone.0310442)
Supplement: S3 File — (DOCX) [file pone.0310442.s004.docx]

Peak area

Peak area


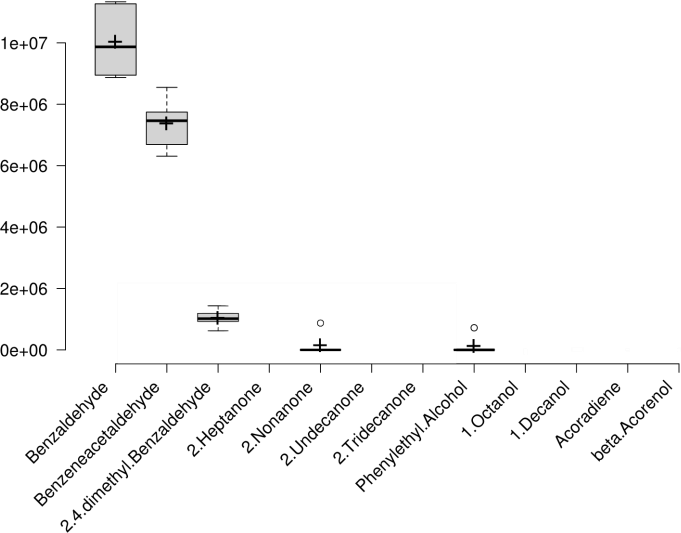


Plain medium volatile profile

Commercial medium


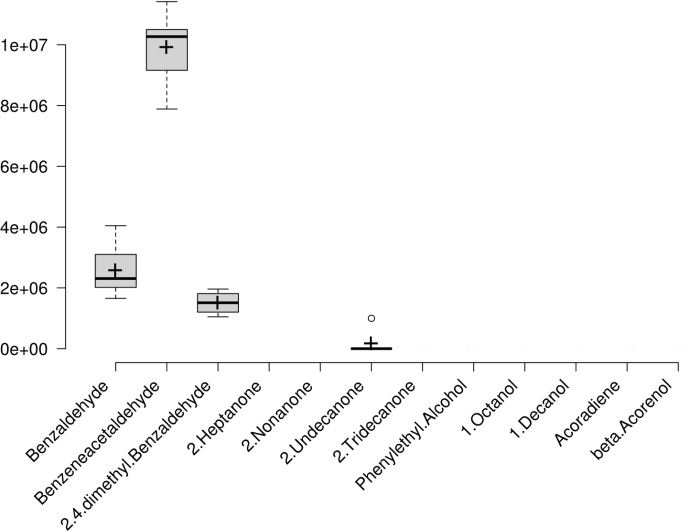


Natural medium

*Supplementary Figure 1.* **Volatile profile of the plain media**. Volatiles of plain commercial and natural media were trapped for 4 hours with PDMS tubes (n = 6 per medium) and analyzed via TD-GC-MS. The shown compounds are specific to the plain media, other compounds that were present in the headspace of plain media and fungal strains were excluded from analysis. Solid lines indicate medians, plus signs means, open circles outliers, boxes the interquartile ranges and whiskers extend up to 1.5 times the interquartile range. Boxplots were generated using [http://boxplot.tyerslab.com.](http://boxplot.tyerslab.com/)

*Phomopsis* volatile profile


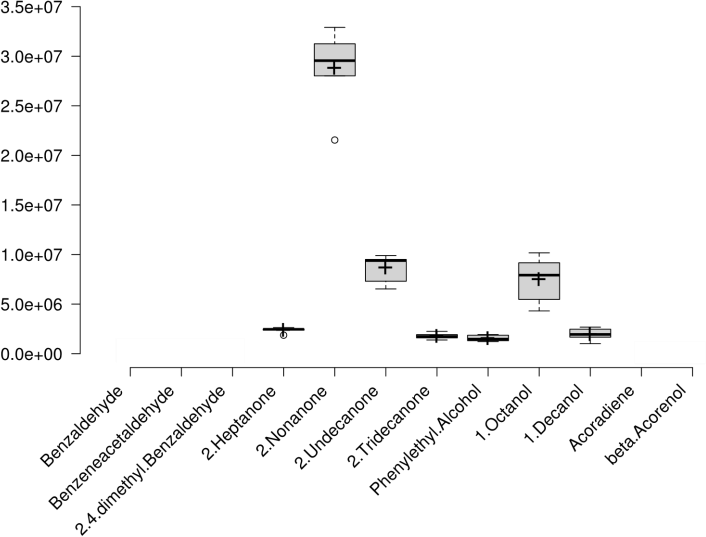

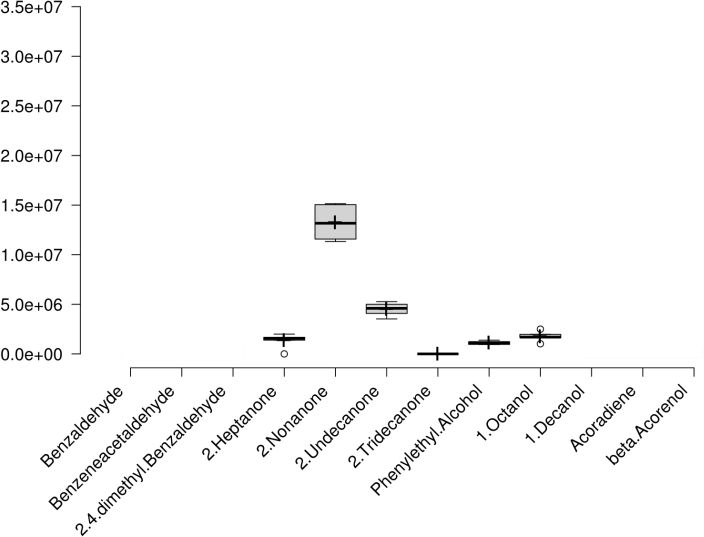


Commercial medium

Natural medium

Peak area

*Supplementary Figure 2.* **Volatile profile of the endophytic strain 2.6 (*Phomopis*) grown on commercial and natural media**. Volatiles were trapped for 4 hours with PDMS tubes (n = 6 per medium) and analyzed via TD-GC-MS. Solid lines indicate medians, plus signs means, open circles outliers, boxes the interquartile ranges and whiskers extend up to 1.5 times the interquartile range. Boxplots were generated using [http://boxplot.tyerslab.com.](http://boxplot.tyerslab.com/)

*Fusarium proliferatum* volatile profile


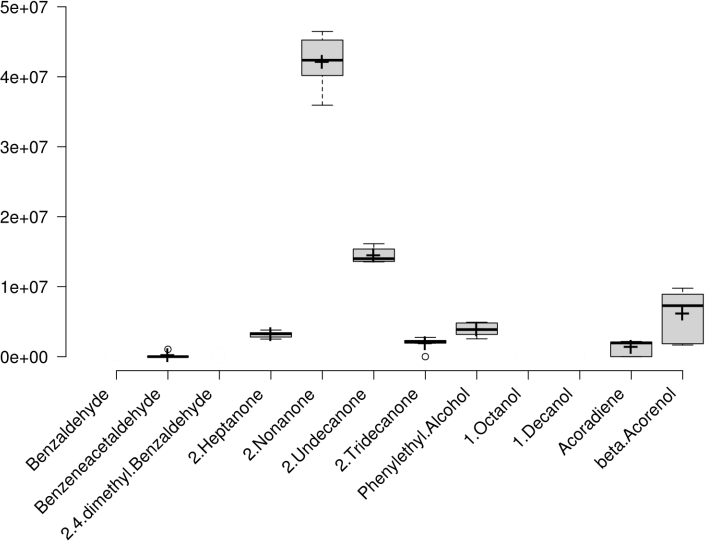


Commercial medium

Peak area


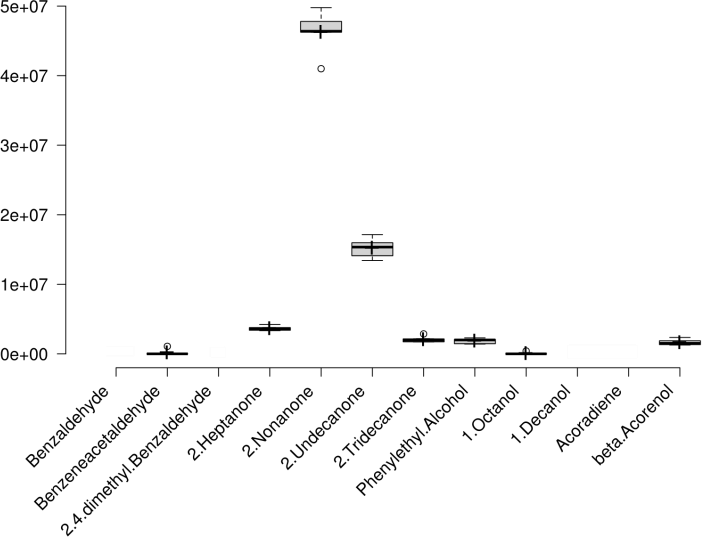


Natural medium

*Supplementary Figure 3.* **Volatile profile of the endophytic strain 8-3 (*F. proliferatum*) grown on commercial and natural media**. Volatiles were trapped for 4 hours with PDMS tubes (n = 6 per medium) and analyzed via TD-GC-MS. Solid lines indicate medians, plus signs means, open circles outliers, boxes the interquartile ranges and whiskers extend up to 1.5 times the interquartile range. Boxplots were generated using [http://boxplot.tyerslab.com](http://boxplot.tyerslab.com/)

*Fusarium graminearum* volatile profile

Peak area


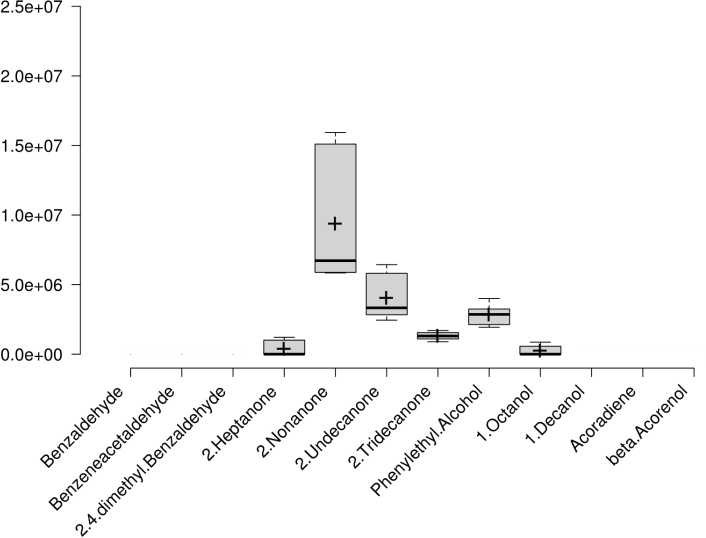


Commercial medium


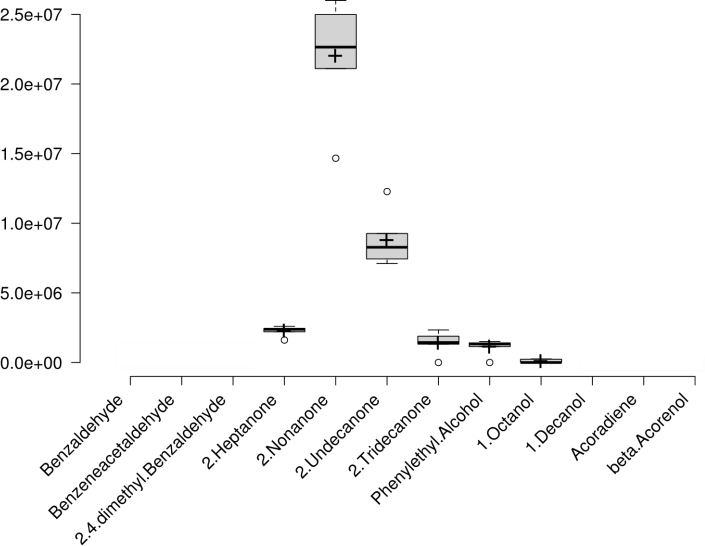


Natural medium

*Supplementary Figure 4.* **Volatile profile of the pathogenic *F*. *graminearum* strain grown on commercial and natural media**. Volatiles were trapped for 4 hours with PDMS tubes (n = 6 per medium) and analyzed via TD-GC-MS. Solid lines indicate medians, plus signs means, open circles outliers, boxes the interquartile ranges and whiskers extend up to 1.5 times the interquartile range. Boxplots were generated using [http://boxplot.tyerslab.com](http://boxplot.tyerslab.com/)
